# Happy or not? An investigative study on well-being and anhedonia in everyday life

**DOI:** 10.1371/journal.pone.0331769

**Published:** 2025-09-11

**Authors:** Sarah A. Merklein, Jutta Peterburs, Annakarina Mundorf

**Affiliations:** 1 Institute for Systems Medicine and Department of Human Medicine, MSH Medical School Hamburg, Hamburg, Germany; 2 Department of Psychology, MSH Medical School Hamburg, Hamburg, Germany; Donald and Barbara Zucker School of Medicine at Hofstra/Northwell, UNITED STATES OF AMERICA

## Abstract

Anhedonia, the inability to experience pleasure or interest in activities, is a key symptom across various psychiatric disorders, including depression. It links to poor quality of life, higher suicide risk, and poorer prognosis. While anhedonia is often studied in clinical populations, its prevalence in healthy individuals and its dimensional nature remain underexplored. This study examined the prevalence of anhedonia in 128 German university students, comparing those with and without psychiatric symptoms and modeling psychiatric symptoms as predictor of anhedonia, measured with the Dimensional Anhedonia Rating Scale (DARS). Results showed that the clinical group demonstrated relatively low levels of anhedonia (total DARS-26: 85.03 ± 12.88) that did not differ from the healthy group (88.80 ± 7.88). Considerable variability suggests that a purely categorical approach to anhedonia may not capture its full complexity. Multiple regression analyses revealed that negative symptoms (e.g., affective flattening) were the strongest predictor of anhedonia, both in the full (b = −0.54, *p* < .001) and within the (sub-)clinical sample (b = −0.64, **p* *< .001). Anxiety was also a significant predictor in the (sub-)clinical group (b = −1.06, **p* *= .01), underscoring its impact on reward processing. Depression did not emerge as a strong predictor (b = −0.21, **p* *= .49) when considered alongside other variables, indicating that its link to anhedonia may be mediated by other factors. Multigroup confirmatory factor analysis of the DARS showed that the 17-item version provided a better fit than the 26-item version, reinforcing the shorter version as a more efficient tool for assessing anhedonia. Taken together, the present findings support the use of a dimensional approach to anhedonia, which offers a more nuanced view of the underlying psychological and neurological mechanisms. Future research should explore anhedonia in larger, diverse samples for a comprehensive understanding of the link between anhedonia and mental illness.

**Registration**: The study was pre-registered on OSF: https://doi.org/10.17605/OSF.IO/234A7.

## Introduction

Anhedonia is a transdiagnostic symptom that can manifest across diverse psychiatric disorders, e.g., major depressive disorder [1–3], schizophrenia [2,4], post-traumatic stress disorder [5], substance abuse disorder [6], and Parkinson’s disease [7]. It refers to a diminishment of pleasure and interest in activities, first described in 1896 by the French psychologist Théodule Ribot [8]. It is associated with a reduced quality of life [9,10], a higher suicide risk [11], and poorer prognosis [12,13]. Research suggests that anhedonia can also occur in individuals without diagnosed psychiatric disorders [14–17], serving as a potential risk factor for various mental illnesses [13,18,19]. The National Institute of Mental Health (NIMH) describes the approach of the Research Domain Criteria (RDoC), which is intended to capture mental disorders based on dimensions of symptom domains and thus complements the symptomatic diagnoses. The RDoC aims to identify different domains that facilitate the transdiagnostic understanding of the constructs and their biological basis in mental illnesses [20]. Herein, anhedonia is a behavioral element of the negative valence system that controls the reactions to aversive contexts and, with the construct of anhedonia, implies a deficit of positive affect. This study aims to address gaps in our understanding of how anhedonia manifests across clinical and non-clinical populations of university students.

### Different types of anhedonia

Treadway and Zald (2011) propose a distinction between consummatory anhedonia (i.e., the inability to experience pleasure from consumption), motivational/anticipatory anhedonia (i.e., the inability to experience pleasure from the anticipation of consumption), and a behavior-modulating/decisional component of anhedonia (i.e., the inability to experience pleasure from the decision to consume) based on underlying neural mechanisms [21]. Der-Avakian and Markou (2012) propose the isolation of the cognitive processes involved, such as stimulus processing and cost-benefit considerations, which are described in the decision-making component by Treadway and Zald (2011) [21,22]. As posited by Treadway and Zald (2011), deficits in positive affect in response to positive stimuli are indicative of consummatory anhedonia. This results in difficulty in deriving pleasure from rewards like eating, drinking, or leisure activities. The reduced anticipation of future rewards or activities that characterizes anticipatory anhedonia often leads to reduced motivation to achieve them. Alterations in the desire to engage in activities and the capacity to derive pleasure from them influence the decision-making processes of individuals with anhedonia. These distinctions are consistent with the constructs of reward anticipation and reward response of the positive valence system as defined by the RDoC [20]. Accordingly, most questionnaires evaluate consummatory and anticipatory pleasure and distinguish between domains like sensory experiences, participating in activities, social interactions, and food/drink consumption [23].

### The DARS and complementary measures of anhedonia

Anhedonia can be assessed using a range of psychometric instruments, each emphasizing different facets of the construct. As the present study applied the Dimensional Anhedonia Rating Scale (DARS), it is important to distinguish between categorical subtypes of anhedonia (e.g., anticipatory, consummatory, decisional) and the dimensional structure reflected in the DARS [23]. Although the categorical subtypes are capable of capturing distinct components of anhedonia, the dimensional structure emphasizes the intensity across all categories. The DARS assesses anhedonia across four hedonic domains, i.e., areas of hedonic experience that can be positive or negative, and that can involve feelings of pleasure or displeasure. The domains utilized are hobbies (e.g., interest and enjoyment in leisure activities such as reading or playing games), food and drink (e.g., pleasure derived from eating or drinking favorite items), social activities (e.g., engagement and enjoyment in interactions with friends or family), and sensory experiences (e.g., appreciation of music, nature, or physical sensations). It does not isolate specific anticipatory or consummatory components of hedonic processing, but instead provides a dimensional evaluation based on self-reported interest, motivation, and enjoyment. The DARS is individualized by asking participants to first identify their favorite activities within each domain. These personally relevant examples then serve as the basis for their ratings, which allows for a more ecologically valid and participant-centered assessment of anhedonic symptoms. Measuring hedonic functioning across these distinct domains allows researchers and clinicians to identify which areas of life are most affected by reduced capacity for pleasure, providing a more nuanced understanding of how anhedonia manifests in daily functioning. This format complements but does not directly align with theoretical models that distinguish between anticipatory, consummatory, and decisional anhedonia.

Other instruments, for example, the Revised Physical Anhedonia Scale (RPAS) [24] and the Revised Social Anhedonia Scale (RPSAS) [25] primarily capture trait-level anhedonia, which refers to a stable, enduring tendency to experience reduced pleasure across various situations and over time [24]. The RPAS focuses on the inability to experience pleasure from sensory or physical stimuli, while the RPSAS assesses diminished pleasure in interpersonal and social contexts. These instruments categorize individuals based on the presence of enduring anhedonic traits; however, they do not differentiate between subtypes of hedonic processing. The Temporal Experience of Pleasure Scale (TEPS) [26], developed within the framework of affective neuroscience, differentiates between anticipatory and consummatory anhedonia [27]. The TEPS’s incorporation of subscales for each category aims to provide a more nuanced understanding of hedonic deficits. However, it is important to note that it does not address specific life domains or personalized preferences. The third subtype, decisional anhedonia, is not explicitly captured by TEPS but has been discussed in more recent theoretical models [22]. The Snaith-Hamilton Pleasure Scale (SHAPS) [28] is another widely used instrument that provides a global measure of hedonic capacity. It is designed to assess the state-level presence of anhedonia, specifically consummatory pleasure, in various domains such as food, social interaction, interests, and sensory experiences. The SHAPS consists of general statements rated by the participant and does not distinguish between anticipatory and consummatory processes, nor does it offer personalized or domain-specific content.

### Mechanisms underlying anhedonia and its predictors

Previous studies have primarily focused on explaining anhedonia symptoms in specific patient groups, predominantly those with depression, as anhedonia is a diagnostic criterion of the disorder according to the Diagnostic and Statistical Manual of Mental Disorders (DSM-V) [29]. Depressive symptomatology is consistently associated with reduced ability to experience pleasure in both anticipatory and consummatory domains, particularly in social and motivational contexts [30,31]. In a meta-analysis comparing patients with major depressive disorder, schizophrenia, substance use disorders, Parkinson’s disease, and chronic pain and healthy individuals, the latter group exhibited lower levels of anhedonia in the SHAPS. Additionally, patients with major depressive disorder showed higher anhedonia when compared with other patient groups in a meta-regression analysis [32]. Furthermore, symptoms of anhedonia are related to anxiety [33,34]. Winer et al. (2018) identified a possible mechanism to explain the relationship between anxiety, depression, and anhedonia. According to their model, anhedonia acts as a mediator for the relationship between anxiety and depression: An individual may develop anxious symptoms, which lead to a loss of interest in rewarding activities, which in turn may result in depressive symptoms. The reverse pattern could also be the case, as loss of interest can lead to anxiety because of the loss and manifest sadness [33,34].

In addition to depression and anxiety, stress has been implicated as a contributing factor in the development and exacerbation of anhedonic symptoms. Chronic stress has been shown to impair reward sensitivity and disrupt dopaminergic signaling, leading to reduced responsiveness to pleasurable stimuli [35]. Studies have shown that prolonged exposure to psychosocial stress can blunt affective reactivity and reduce motivation to pursue rewarding experiences, even in individuals without clinical depression [36,37]. Especially university students experience high stress levels [38] associated with different psychiatric symptoms [39].

However, only a few studies have reported on the prevalence of anhedonia in healthy adults [14,16,17]. For instance, the occurrence of anhedonia in healthy individuals was confirmed in a sample of students in Beijing [14]. Anhedonia was operationalized with the RPAS, RPSAS, and TEPS. Prevalence was calculated by counting each item that indicated anhedonia and ranged from 4.4 to 89.4%. More pronounced anhedonia was found in females compared to males. Another study conducted in a sample of healthy Italian adults reported that 14.9% experienced anhedonia when the cut-off values of the SHAPS were considered. No link between age or gender was found with SHAPS scores [16]. Wellan et al. (2021) examined the effects of the COVID-19 pandemic on state anhedonia and depression in a healthy sample using the DARS [17]. Slightly elevated scores for female compared to male participants were found, while no effect of age was observed. However, findings on gender differences in anhedonia remain inconsistent across studies. Some studies have reported higher levels of anhedonia in healthy women [14,17] and women with depression [40] or no significant differences [16], while another study has found greater deficits in men with schizotypy [41].

### Study aims

The present study aimed to investigate the prevalence and distribution of anhedonia symptoms among German students, comparing those with and without clinically noticeable psychiatric symptoms. University students are considered a relevant population for the investigation of subclinical symptomatology due to both methodological and clinical reasons. The relative homogeneity in age, educational background, and life context allows for greater control over confounding variables, which can improve internal validity [42,43]. This homogeneity is particularly beneficial when studying subclinical symptom expressions, such as anhedonia, within dimensional frameworks. At the same time, this population is increasingly affected by mental health problems. Elevated rates of depression [44] and stress [38] have also been documented in student populations relative to the general population. These factors make university students particularly suitable for examining the early and subthreshold manifestations of symptoms such as anhedonia within a dimensional research framework.

The primary focus of the study was to assess the severity and variability of anhedonia using the Dimensional Anhedonia Rating Scale (DARS) [23], and to explore its relationship with other psychiatric symptoms across both healthy and clinically affected individuals. A further aim was to evaluate the utility of a dimensional approach to understanding anhedonia and its associations with other psychiatric symptoms. This approach could offer new insights into the transdiagnostic nature of anhedonia and its relevance across a spectrum of psychiatric conditions.

Finally, to examine the psychometric robustness of the DARS in this population, the study employed multigroup confirmatory factor analysis (CFA) to evaluate whether the instrument validly captures anhedonia symptoms across individuals with and without clinical symptomatology.

## Methods

### Subjects and recruitment

Participants were recruited through the MSH Medical School Hamburg SONA system (Sona Systems; https://www.sona-systems.com), an online participant pool management system for universities. Further advertising was carried out through flyers in the university. Inclusion criteria required an age of 18–35 years and current enrollment at MSH Medical School Hamburg. Participants over 35 were excluded to maintain focus on a university-student population before entering middle age as defined by the American Psychological Association [45]. The survey was accessible online via the Unipark platform (www.unipark.de) and could be filled in on a smartphone or computer. After reading the study description, participants gave their written informed consent online. A total of four questionnaires were included, which took a maximum of 30 minutes (mean time for completion 18 minutes). Participation was anonymous, voluntary, and it was possible to discontinue the study at any time without any disadvantage to the participants. Participation was rewarded with course credit (1 credit = 1 hour). Data collection took place during March, April, and May 2024. The study procedures conformed to the Declaration of Helsinki and received ethical approval (MSH-2024/305) from the Ethics Board of the MSH Medical School Hamburg, Germany. Data and code are available at https://osf.io/djmr7 (Merklein, Peterburs, Mundorf, 2025).

### Inventories

Anhedonia was measured as dependent variable operationalized by the Dimensional Anhedonia Rating Scale (DARS) [23]. The Motivation and Pleasure Scale – Self-Report (MAP-SR) [46] served as a screening for negative symptoms in psychotic disorders, including facets of anhedonia (e.g., loss of motivation). Furthermore, depressive symptoms, as well as symptoms of anxiety, were surveyed with the Depression-Anxiety-Stress Scales (DASS) [47]. Participants were screened for potential psychiatric disorders with the ICD-10 Symptom Rating (ISR) [48].

The German translation of the 26-item version of the DARS (by A. Bieda, Ruhr-University Bochum, Germany, retrieved from: https://osf.io/gm4fw/) was applied to ensure comprehensive coverage of all hedonic dimensions including interest, motivation, effort, and pleasure during consumption in the areas of hobbies (9 items, e.g., “I would have a desire to participate in these activities.”), eating/drinking (6 items, e.g., “Having these foods/drinks would satisfy me.”), social activities (6 items, e.g., “Spending time doing these things would make me happy.”), and sensory experiences (5 items, e.g., “I get excited thinking about these experiences.”). Rizvi et al. (2015) and Wellan et al. (2021) confirmed the four-factor structure for the original 17-item version as well as good validity and high internal consistency across both patient samples (α = 0.96) and the general population (α = 0.92) [17,23]. The extended 26-item version stems from adding 9 items to measure motivational aspects [17,49]. For each area, participants name two to three favorite examples to which the following items refer. Therefore, the scale takes individual preferences and cultural beliefs into account. The item statements are classified on a five-point scale (0 “not at all” – 4 “very much”) with higher sum scores indicating higher anhedonia symptomatology.

The MAP-SR [46] was used to assess negative symptoms. It consists of 15 items measuring motivation, effort, and interest across various areas of life on a five-point scale ranging from 0 (“no pleasure “) to 4 (“extreme pleasure”). The items (e.g., “In the past week, what is the most pleasure you experienced from being with other people?”) are inverted, meaning higher values indicate more pronounced negative symptoms. The MAP-SR demonstrates adequate internal consistency, with a reliability coefficient of α = 0.88 [50]. Additionally, the German version of the MAP-SR demonstrated comparable convergent and discriminant validity to the American version. While both the DARS and MAP-SR assess aspects of anhedonia, the DARS captures domain-specific subjective pleasure, whereas the MAP-SR focuses on motivation and pleasure as part of negative symptomatology. Including both allows for a more nuanced differentiation of hedonic experience and functional motivation.

The DASS [47,51] captures psychological symptoms of depression (7 items, e.g., “I found it difficult to work up the initiative to do things.”), anxiety (7 items, e.g., “I was worried about situations in which I might panic and make a fool of myself.”), and stress (7 items, e.g., “I found it difficult to relax.”). The item statements refer to the past week, and response options range from 0 (“did not apply to me at all”) to 3 (“applied to me very much or most of the time”). Scale-specific total scores are obtained, which are compared with cut-off values. There is an increased probability of a depressive disorder with sum scores of 10 or higher, for anxiety disorders from 6 or higher, the cut-off value for the stress scale is 10. The internal consistency of the scale was determined to be Cronbach’s α = .91 for depression, .78−.82 for anxiety, and .81−.89 for stress [47].

Upon survey completion, participants were asked to fill in the ISR [48,52]. This rating served as a standardized diagnostic instrument intended to allow for conclusions to be drawn about possible psychiatric disorders. The ISR is a screening instrument for psychiatric disorders and queries depression (4 items, e.g., “I no longer enjoy doing things I used to enjoy.“), anxiety (4 items, e.g., “I try to avoid these harmless frightening situations.“), compulsion disorder (3 items, e.g., “I try to resist recurring, seemingly senseless thoughts and actions, but often don’t succeed.“), somatization (3 items, e.g., “I feel the need to see a doctor about inexplicable physical problems.“), and eating disorder (3 items, e.g., “I spend a lot of time thinking of ways to lose weight.“). An additional scale (12 items) measures concentration disorders, suicidal tendencies, sleep problems, everyday stress, trauma, and sexuality (e.g., “I suffer from recurring dreams or flashbacks of horrible events.“). The symptoms are rated on a scale from 0 (“does not apply”) to 4 (“applies extremely”). In the evaluation, the mean values of the scales are calculated, and an ISR total score is obtained as the mean value of all scales, with double weighting of the additional scale because it encompasses a broader range of domains (concentration, suicidality, sleep, stress, trauma, sexuality). Scale-specific cut-off values provide an orientation for the classification of symptom severity (“suspected” to “severe symptom burden”). Cronbach’s alpha indicated high reliability for the total score (α = 0.90) and acceptable to good reliability for the subscales: somatoform symptoms (α = 0.82), phobic anxiety (α = 0.85), depressive symptoms (α = 0.80), obsessive-compulsive symptoms (α = 0.78), and eating disorders (α = 0.81) [53].

### Pre-registration

The study was pre-registered on OSF.io prior to any human observation of the data (https://doi.org/10.17605/OSF.IO/234A7). The time-stamped date of registration in OSF.io was April 17^th^, 2024. Pre-registration was conducted to ensure unbiased data analysis.

### Deviations from the pre-registered protocol

Deviation from the pre-registered protocol occurred for the software used for data analysis: R Studio was used instead of JASP. To evaluate the ability of individual clinical measures to discriminate between participants classified as healthy (Group 0) and those with subclinical symptoms (Group 1) and based on the predicted probabilities, receiver operating characteristic (ROC) curves were generated, and the area under the curve (AUC) was calculated for each mode (pROC package [54]). AUC was used as a metric for classification accuracy, with higher values indicating better discriminative performance [55]. Additionally, due to violation of the normality and homoscedasticity assumptions, outliers were excluded from the multiple linear regression analysis in accordance with the criteria set forth by Cook (1977) that any observation with a Cook’s distance value exceeding four times the sample size (n) should be excluded [56]. An exploratory repeat of the multiple linear regression analysis was performed including all scales to identify specific predictors regarding the psychiatric symptoms as they showed more deviation across the sample.

### Statistical analysis

The G*Power software program [57] was used to conduct *a priori* power analysis. To detect a small effect size of f = 0.15 (using linear multiple regression, fixed model, R2 deviation from zero) with an alpha error probability of .05, a power of .95, and four predictors, a total sample size of 129 participants is required.

The statistical analyses were carried out using R Statistical Software (version 4.4.0 [58]) with RStudio (version 2023.12.1 [59]).

Two groups were formed based on (sub-)clinical characteristics: The first group comprised healthy participants (Group 1, hereinafter referred to as ‘healthy group’). This group included participants who neither reported the presence of a psychiatric disorder nor exhibited scores above cut-off values of the DASS (applying the cut-offs of the depressive and anxiety scales indicates an increased likelihood of the presence of depressive and anxiety disorders, according to the scoring manual) or the ISR (applying the cut-off values from the evaluation manual for an average symptom burden; cut-off depression = 2, cut-off anxiety = 2, cut-off OCD = 2, cut-off somatoform = 1.33, cut-off eating disorder = 1.67). The subclinical/clinical group (Group 2, hereinafter referred to as ‘clinical group’) comprised participants who reported the presence of a psychiatric disorder and those who exceeded the cut-off values of the DASS or the ISR (as described above). ROC analyses were applied to assess the specificity of each grouping variable. Outliers were included in the confirmatory factor analysis.

First, a descriptive analysis was conducted to evaluate the severity of anhedonia and the distribution of the scores for MAP-SR, DASS, and ISR ICD-10. Skewness and kurtosis were examined across all scales. Next, multiple linear regression was performed with the DARS score as the dependent variable and the MAP-SR score, DASS anxiety and depression scores, and mean total ISR score as independent variables. Exploratory analyses included a second multiple linear regression with all scales serving as independent variables, as well as a third model for the clinical group only. The following tests were conducted to assess the assumptions underlying the analysis: the Rainbow Test for linearity (lmtest package v0.9-38 [60]), the Shapiro-Wilk Test for normality, the Durbin-Watson Test for autocorrelation (car package v3.1-3 [61]), and the Breusch-Pagan Test for homoscedasticity (lmtest package v0.9-38 [60]), the Variance Inflation Factor (VIF) for multicollinearity (car package v3.1-3 [61]). Prior to regression analysis, multicollinearity between DARS and MAP-SR, as well as the other inventories, was assessed (all VIF values < 5), suggesting acceptable levels of overlap.

We calculated the Akaike Information Criterion (AIC) and Bayesian Information Criterion (BIC) values to identify the optimal model and to perform comparison analyses. The comparisons were conducted separately for the models incorporating the entire sample and for those focused on the clinical group.

To determine if the extended 26-item DARS appropriately measured anhedonia in both students meeting cut-off scores for psychiatric disorders and healthy students, we conducted a multigroup confirmatory factor analysis (multigroup CFA) of the four-factor model, which differentiates four hedonic domains: hobbies, food/drinks, social activities, and sensory experiences. Multigroup CFA was performed using the lavaan package v0.6-18 [62]. To gain further insight and facilitate a comparison of the two versions of the DARS, another multigroup CFA was conducted for the 17-item version of the DARS. This was achieved by excluding the additional items from the 26-item German version.

## Results

### Sample description and grouping based on screening

A total of 129 students completed the survey. One subject did not meet the inclusion criteria regarding age (was older than 35 years) and was excluded from further analysis, resulting in a total sample size of 128 (mean age 23.74, 21.9% male). Sixty-one participants (15 males) were included in the healthy group. In comparison, 76 participants (13 males) reported the presence of a psychiatric disorder or exceeded cut-off values from the DASS or ISR, forming the clinical group. There were no significant differences between the groups with respect to age (*p = *.88), gender (*p = *.62), relationship status (*p = *.77), or educational training (*p = *.41). The healthy group was medication- and psychotherapy-naïve, whereas the clinical group comprised 18 individuals undergoing psychotherapy and 13 subjects taking medication. The most prescribed psychiatric medication was antidepressants (n = 10), while other subjects were administered lisdexamfetamine (n = 1), Ritalin (n = 1), and Quetiapine (n = 1). The demographic characteristics are presented in Table 1.

**Table 1:** Demographic group characteristics.

	Total sample (n = 128)	Group 1 (healthy) (n = 61)	Group 2 (clinical) (n = 67)	t-test p-value
Age	23.74 ± 3.27	23.79 ± 3.54	23.7 ± 3.03	.88
Gender (m/f/d)	28/100/0 (21.88%)	15/46/0 (24.59%)	13/54/0 (19.4%)	.62 (Chi^2^)
Children (y/n)	3/125 (2.34%)	1/61 (1.64%)	2/65 (2.99%)	
Psychiatric diagnosis (y/n)	25/103 (19.53%)	0/61	25/42 (37.31%)	
Psychotherapy	18/110 (14.06%)	0/61	18/49 (26.87%)	
Medication	13/115 (10.16%)	0/61	13/54 (19.4%)	
Relationship status				.77
single	63	31	32	
in a relationship	60	27	33	
married	5	3	2	
Education				.41
A-level	60	25	35	
Bachelor’s degree	61	32	29	
Master’s degree	3	1	2	
Vocational training	4	3	1	

Groups were formed using self-reported psychiatric disorders, the DASS (depression and anxiety sub-scores), and ISR (all scales) cut-off values.

In order to examine the discriminative power of individual clinical variables in identifying participants with clinical symptoms, a series of ROC analyses were conducted. Given that the grouping variable was derived based on thresholds from the included predictors, the results of the ROC should be interpreted with caution, as they reflect the operational criteria used to define subclinical status. It is imperative to acknowledge that the outcomes of these analyses are intended to serve as a descriptive reflection of the operational criteria employed to define groups. The findings indicated that the sum scores for anxiety (DASS) achieved the highest classification accuracy (AUC = 0.833), followed by the ISR anxiety (AUC = 0.824), the ISR depression (AUC = 0.796), and DASS depression (AUC = 0.777). The other variables demonstrated moderate discriminative ability, including ISR OCD (AUC = 0.747), ISR eating disorder (AUC = 0.727), and ISR somatoform (AUC = 0.676). These findings suggest that affective symptom dimensions, particularly anxiety and depression, were the most effective in differentiating between healthy and clinical participants.

### Prevalence of anhedonia and psychiatric symptoms

The results from the questionnaires are summarized in Table 2. Groups showed significant differences in all scales utilized for their formation, with the clinical group displaying higher symptomatology (*p < *.001). Furthermore, the clinical group exhibited significantly elevated scores on the DASS stress subscale (*p < *.001), the MAP-SR (*p < *.001), and increased anhedonia as indicated by reduced DARS scores regarding the activity (*p < *.01) and social (*p = *.01) domains. Marginal between-group differences were observed in the total score (*p = *.05), and no differences in the subscales of consummatory pleasure (*p = *.51) and sensory experience (*p = *.62). Supplementary S1 Table presents representative examples of individual preferences provided by participants in the DARS. A correlation matrix for the scales is provided in the supplementary materials S2 Fig.

**Table 2:** Group characteristics regarding clinical inventories.

	Total sample (n=128)	Group 1 (healthy) (n=61)	Group 2 (clinical) (n=67)	t-test p-value
*DARS (26-item)*				
total	86.83 ± 10.92	88.80 ± 7.88	85.03 ± 12.88	.05
activity	31.41 ± 4.22	32.41 ± 3.07	30.49 ± 4.90	.009*
consumption	19.03 ± 4.27	19.29 ± 4.13	18.79 ± 4.41	.51
sensory experience	16.92 ± 2.89	16.79 ± 2.82	17.04 ± 2.98	.62
social	19.47 ± 3.68	20.31 ± 2.80	18.70 ± 4.21	.01*
*DARS (17-item)*				
total	56.80 ± 7.14	57.71 ± 5.38	55.99 ± 8.38	.17
activity	14.19 ± 1.95	14.52 ± 1.46	13.88 ± 2.28	.06
consumption	12.88 ± 2.80	13.05 ± 2.67	12.72 ± 2.93	.50
sensory experience	16.92 ± 2.90	16.79 ± 2.82	17.04 ± 2.97	.62
social	12.82 ± 2.49	13.34 ± 1.86	12.34 ± 2.88	.02*
*MAP-SR*	19.67 ± 7.61	17.28 ± 6.71	21.85 ± 7.78	<.001**
*DASS*				
depression	4.64 ± 4.73	2.31 ± 2.23	6.76 ± 5.38	<.001***
anxiety	3.99 ± 3.81	1.77 ± 1.56	6.01 ± 4.12	<.001***
s tress	6.45 ± 4.23	4.49 ± 3.14	8.22 ± 4.32	<.001***
*ISR*				
total	0.76 ± 0.59	0.36 ± 0.23	1.13 ± 0.59	<.001***
depression	1.1 ± 0.93	0.6 ± 0.46	1.56 ± 1	<.001***
anxiety	0.96 ± 0.9	0.45 ± 0.46	1.42 ± 0.92	<.001***
OCD	0.72 ± 0.82	0.34 ± 0.52	1.07 ± 0.89	<.001***
somatoform	0.41 ± 0.72	0.14 ± 0.26	0.66 ± 0.89	<.001***
eating disorder	0.84 ± 1.06	0.36 ± 0.52	1.27 ± 1.23	<.001***
additional scale	0.64 ± 0.58	0.31 ± 0.26	0.95 ± 0.61	<.001***

Groups were formed using self-reported psychiatric disorders, the DASS (depression and anxiety sub-scores), and ISR (all scales) cut-off values. *DARS:* Dimensional Anhedonia Rating Scale; DASS: Depression Anxiety Stress Scales; MAP-SR: Motivation and Pleasure Scale – Self-Report; ISR: ICD-10-Symptom-Rating.

### Modeling anhedonia severity from symptom dimensions

Following the removal of outliers in accordance with the criteria set forth by Cook (1977) [56], the analysis provided evidence to support the validity of the model’s underlying assumptions. There was no evidence of a violation of linearity (Rainbow Test; *R = *0.91, *p = *.63). Results of the Shapiro-Wilk test indicated that the residuals were normally distributed (*W =* 0.98, *p = *.11), and the Durbin-Watson test confirmed the absence of significant autocorrelation (*D =* 1.69, *p = *.10). The Breusch-Pagan test yielded a confirmation of homoscedasticity (*BP =* 5.31, *p = *.26). With the exception of the ISR somatoform subscale (skewness = 2.42), all skewness values fell below 2, indicating acceptable symmetry. Kurtosis values remained under 7 for every measure; only three scales showed moderate leptokurtosis (DARS total = 5.20, DARS social = 3.81, ISR somatoform = 6.74). Furthermore, the VIF values for the predictors indicated the absence of strong multicollinearity (VIF < 5) with values ranging from 1.55 (MAP-SR) to 2.73 (DASS depression). A significant regression equation was found (R²Adjusted = .259, F_(4,114)_ = 11.29, *p < *.001), results are presented in Table 3. Among the predictors, the negative symptoms measured with the MAP-SR were found to significantly predict anhedonia (b = −0.490, t_(114)_ = −4.402, *p < *.001). Additionally, the mean total score of the ISR was significant as a predictor of anhedonia (b = 3.71, t_(114)_ = 2.057, *p = *.04). DASS depression and DASS anxiety did not significantly predict anhedonia (s. Table 3). Depression, as measured by the DASS, was a significant predictor when used as a standalone variable in an exploratory hierarchical regression (b = −0.68, *p < *.001). Statistical significance diminished when the other predictors were added.

**Table 3:** Results from multiple linear regression analysis.

Predictor	Dependent variable: anhedonia (DARS)							
	Unstandardized coefficients		Z-Standardized coefficients				Collinearity	
	*b*	*SE b*	*b*	*SE b*	t	*p*	*Tolerance*	*VIF*
(Intercept)	97.18	2.04	−2.227e-16	7.893e-02	47.538	<2e-16		
DASS anxiety	−.41	.26	−1.767e-01	1.155e-01	−1.530	.13	.471	2.123
DASS depression	−.28	.26	−1.382e-01	1.309e-01	−1.056	.29	.367	2.727
MAP-SR	−.49	.11	−4.341e-01	9.862e-02	−4.402	<.001***	.646	1.548
ISR additional scale	3.71	1.80	2.451e-01	1.192e-01	2.057	.04*	.442	2.260
*Multiple R* ^2^ *Adjusted R* ^2^	.2838 .2586			<.001***				
*F*	11.29 (4 and 114 DF)							
*Residual SE*	6.817							

*DARS* Dimensional Anhedonia Rating Scale; DASS: Depression Anxiety Stress Scales; MAP-SR: Motivation and Pleasure Scale – Self-Report; ISR: ICD-10-Symptom-Rating; VIF: Variance Inflation Factor.

In addition to the primary analysis with four predictors, exploratory replication of the regression model with all measured scales as predictors was conducted (Table 4). After removing outliers, assumption checks confirmed no violations: linearity was supported (Rainbow Test; *R = *.96, **p* *= .56), normality of residuals was indicated (Shapiro-Wilk; *W =* 0.98, *p = *.051), and the Durbin-Watson showed no significant autocorrelation (*D =* 1.79, *p = *.21). The Breusch-Pagan test confirmed homoscedasticity (*BP =* 10.94, *p = *.36), and the VIF values ranged from 1.34 (ISR eating disorder) to 4.28 (ISR depression), indicating the absence of strong multicollinearity (VIF < 5).

**Table 4:** Results from multiple linear regression analysis including all scales.

Predictor	Dependent variable: anhedonia (DARS)							
	Unstandardized coefficients		Z-Standardized coefficients				Collinearity	
	*b*	*SE b*	*b*	*SE b*	t	*p*	*Tolerance*	*VIF*
(Intercept)	97.34	2.12	4.065e-16	.079	45.976	<.001***		
DASS anxiety	−.69	.35	−.303	.154	−1.971	.05	.268	3.727
DASS depression	−.21	.30	−.112	.162	−.689	.49	.241	4.146
DASS stress	.29	.26	.141	.127	1.107	.27	.393	2.541
MAP-SR	−.54	.12	−.485	.106	−4.581	<.001***	.567	1.765
ISR depression	1.02	1.59	.106	.165	.643	.52	.233	4.284
ISR anxiety	1.24	1.33	.135	.145	.934	.35	.302	3.304
ISR OCD	1.81	1.05	.174	.101	1.723	.08	.621	1.608
ISR somatoform	1.03	1.20	.089	.103	.862	.39	.601	1.662
ISR eating disorder	−.12	.77	−.014	.092	−.156	.87	.745	1.341
ISR additional scale	−1.97	2.10	−.131	.141	−.936	.35	.321	3.106
*Multiple R* ^2^ *Adjusted R* ^2^	.3077 .2442				<.001***			
*F*	4.846 (10 and 109 DF)							
*Residual SE*	6.855				0.8694			

*DARS* Dimensional Anhedonia Rating Scale; DASS: Depression Anxiety Stress Scales; MAP-SR: Motivation and Pleasure Scale – Self-Report; ISR: ICD-10-Symptom-Rating; VIF: Variance Inflation Factor.

The adjusted model yielded an adjusted R² of .244 with a significant overall model fit (F_(10,109)_ = 4.846, *p < *.001). Among the predictors, negative symptoms, as measured by the MAP-SR, continued to significantly predict anhedonia (b = −0.54, t_(109)_ = −4.581, *p < *.001). Additionally, anxiety operationalized with the DASS scale exhibited a trend towards significance as a predictor of anhedonia (b = −0.69, t_(109)_ = −1.971, *p = *.051), as well as ISR OCD scores (b = 1.82, t_(109)_ = 1.723, *p = *.088). Other predictors, including DASS depression, DASS stress, and several ISR scales (depression, anxiety, somatoform, eating disorder, additional scale), did not significantly predict anhedonia (s. Table 4).

Subsequently, another model was fitted including gender as a predictor to examine potential gender-related discrepancies and to ascertain the extent to which it contributes to the model’s predictive capacity. The extended model resulted in an adjusted R² of .352 (F_(11,107)_ = 6.833, *p < *.001), with gender identified as a significant predictor (b = 5.12, t_(107)_ = 3.659, *p = *.001). A *post-hoc* t-test revealed that men exhibited higher anhedonia symptomatology (t = −2.69, df* *= 51.97, **p* *< .001) when assessed with the total DARS score (mean = 88.03) compared to women (mean = 82.54) in the whole sample. Please see Supplementary S3 Table for detailed model results.

All models calculated based on the entire sample were evaluated using AIC and BIC. Lower values indicate a superior model fit. The model comprising only four scales yielded an AIC of 942.09 and BIC of 959.20, whereas the model incorporating all scales exhibited a superior fit (AIC = 814.99, BIC = 848.44). The gender-inclusive model provided the best fit (AIC = 785.23, BIC = 821.36).

To determine the influence of the screening, a separate regression model was conducted for the clinical group alone (for results, see Supplementary S4 Table). The adjusted model yielded an adjusted R² of .332 and a significant overall model fit (F_(10,51)_ = 4.033, *p < *.001). Among the predictors, negative symptoms, as measured by the MAP-SR, continued to significantly predict anhedonia in the clinical group (b = −0.64, t_(51)_ = −3.435, *p = *.001), consistent with the results of the full sample analysis. Anxiety (DASS), which exhibited a trend toward significance in the full model, became a significant predictor within the clinical group (b = −1.06, t_(51)_ = −2.649, *p = *.011). Other predictors, such as the DASS depression or stress scales and various ISR subscales (including depression, OCD, somatoform, and eating disorder scales), did not demonstrate significant effects within this group. Notably, the previously observed marginal significance for the ISR OCD scores in the full sample was not replicated in the clinical group (b = 1.62, *p = *.240).

Subsequent exploratory analyses were conducted to investigate the association of anhedonia with self-reported diagnosis, current psychotherapy, and the intake of psychiatric medication in the clinical group. This was achieved by incorporating these variables into the previously described multiple linear regression model (adjusted R² = .377, F_(13,44)_ = 3.654, *p < *.001). The results indicated that neither a self-reported diagnosis (b = 0.07, *p = *.981), nor current psychotherapy (b = −0.63, **p* *= .822), nor the intake of psychiatric medication (b = 2.30, **p* *= .548) were a significant predictor of anhedonia symptomatology when added to the model (for detailed results, see Supplementary S5 Table).

Model comparisons for the clinical group revealed that the model incorporating psychiatric factors (self-reported diagnosis, current psychotherapy, intake of psychiatric medication) exhibited a superior fit, with an AIC of 393.07 and BIC of 423.98, compared to the model without these factors (AIC = 433.85, BIC = 459.37).

### Model comparison of the 26-item vs. 17-item DARS version

The general factor analysis indicated a moderate fit of the general four-factor structure for the 26-item version (CFI = .836, TLI = .818, RMSEA = .082 [90% CI = .072, .093], SRMR = .074, AIC = 6391, BIC = 6556.8). The 17-item version yielded a superior fit of the four-factor structure (CFI = .912, TLI = .894, RMSEA = .067 [90% CI = .047, .085], SRMR = .072, AIC = 4308.1, BIC = 4422.2) compared to the 26-item version. Measurement invariance was established at the configural, metric, scalar, and strict levels, as shown in Table 5.

**Table 5:** Measurement invariances for the MCFA.

Model	*χ2*	*df*	*CFI*	*TLI*	*RMSEA [90% CI]*	*SRMR*
**DARS 26-item version**						
General model	547.56	293	.836	.818	.082 [.072,.093]	.074
Configural	1019.63	586	.745	.717	.108 [.096,.118]	.095
Metric	1089.06	608	.717	.697	.111 [.100,.122]	.114
Scalar	1118.57	630	.713	.703	.110 [.100,.121]	.116
Strict	1184.89	656	.689	.692	.112 [−102,.122]	.124
**DARS 17-item version**						
General model	177.68	113	.912	.894	.067 [.047,.085]	.072
Configural	309.58	226	.894	.873	.076 [.054,.096]	.084
Metric	367.26	239	.838	.815	.092 [.073,.110]	.109
Scalar	391.06	252	.824	.810	.093 [.074,.110]	.112
Strict	438.381	269	.786	.783	.099 [.082,.116]	.123

*DARS* Dimensional Anhedonia Rating Scale.

The chi-squared difference test for the 26-item version (Table 6) indicated that there were significant differences in model fit as the constraints were increased. The configural invariance model, which tests whether the overall factor structure is equivalent across groups, demonstrated a poor fit (χ²_(586)_ = 1019.625, CFI = .745, TLI = .717, RMSEA = .108 [90% CI = .096,.118], SRMR = .095). Results indicate that the four-factor structure of hobbies, food/drinks, social activities, and sensory experiences was consistent across both groups without imposing any equality constraints on the parameters. A statistically significant change in the chi-square value was observed between the metric invariance model, which constrains factor loadings to be equal across groups, and the configural model (Δχ²_(22)_ = 69.435, *p < *.001). Subsequent testing revealed that the chi-squared value (χ²_(608)_ = 1089.060) was elevated and the CFI (CFI = .717) decreased (TLI = .697, RMSEA = .111 [90% CI = .100,.122], SRMR = .114), suggesting the absence of discrepancies in factor loadings between the two groups. The scalar invariance model, which constrains both factor loadings and item intercepts to be equal across groups, did not result in a statistically significant deterioration of model fit compared to the metric model (Δχ²_(22)_ = 29.506, p > .05). This model demonstrated a comparable fit to the metric model (χ²_(630)_ = 1118.566, CFI = .713, TLI = .703, RMSEA = .110 [90% CI = .100,.121], SRMR = .116). The strict invariance model, which additionally constrains residual variances to be equal across groups, resulted in a significant decrease in model fit (Δχ²_(26) =_ 66.331, χ²_(656)_ = 1184.896, CFI = .689, TLI = .692, RMSEA = .112 [90% CI = .102,.122], SRMR = .124). Chi-squared difference tests for the 17-item version yielded comparable results (Table 6).

**Table 6:** Results from the χ2 difference test.

Model	*AIC*	*BIC*	*Model comparison*	*Δχ2*	*RMSEA*	*p*
**DARS 26-item version**						
Configural	6400.8	6879.9	–	–	–	–
Metric	6426.2	6842.6	Configural	69.435	.183547	<.001***
Scalar	6411.7	6765.4	Metric	29.506	.073013	.131
Strict	6426.0	6705.5	Scalar	66.331	.155683	<.001***
**DARS 17-item version**						
Configural	4278.3	4603.4	–	–	–	–
Metric	4310.0	4698.0	Configural	57.683	.23175	<.001***
Scalar	4307.8	4558.8	Metric	23.801	.11394	.03*
Strict	4321.1	4523.6	Strict	47.318	.1669	<.001***

## Discussion

The present study investigated the occurrence of anhedonia in a typical student population and aimed to probe the potential for a dimensional investigation of anhedonia rather than a categorical distinction as a means of elucidating the underlying mechanisms. To this end, healthy individuals and subjects meeting the clinical criteria for diagnosis of a mental illness were included. Based on the results, four principal conclusions can be drawn regarding the prevalence of anhedonia, the role of negative symptoms in the context of anhedonia, the contribution of anxiety to anhedonia in clinical populations, and the validity of the 26-item DARS scale.

### Addressing anhedonia in a university student population

Mean total DARS scores in healthy students were comparable to those reported in previous studies of healthy general populations [17,23,63]. The clinical student group exhibited fewer anhedonia symptoms, as reflected by higher scores (mean total DARS score: 55.99) than those observed in previous clinical studies on the general population, which reported mean scores of 51.2 in MDD patients and 30.2 in patients experiencing a depressive episode (N = 52) [23], along with mean scores of 37.3 in a Polish sample (N = 49) [63]. The current study’s use of screening questionnaires to identify participants with clinically relevant symptomatology may have contributed to the observed results, as subjects may exhibit fewer psychiatric symptoms than those with confirmed diagnoses. Additionally, in the present study, the clinical group was not limited to individuals with depression; groups were formed based on the scales for depression, anxiety, OCD, eating disorders, and somatization. Consequently, the sample did not exclusively consist of officially diagnosed depressed patients and aimed to investigate the prevalence and severity of anhedonia in a mixed sample. Although the cut-off values used for grouping demonstrated good discriminative ability in the ROC analyses, they should not be interpreted as a diagnostic threshold. DSM-5 criteria include multiple facets which may be underrepresented in screening questionnaires. Therefore, while the data support the threshold’s utility for screening purposes, its clinical generalizability should be interpreted with caution.

Lin et al. (2022) proposed a threshold of 28.5 to identify significant anhedonia symptomatology in MDD patients with the DARS [64]. In our healthy sample, no individual scored less than 44 points on the total scale. The lowest score was 24, and only two individuals scored lower than the proposed threshold for clinically significant anhedonia symptoms. These results indicate that the proposed threshold did not capture a sufficiently broad range and degree of variation in anhedonia symptoms within our student sample, even with individuals meeting clinical cut-off scores included. Our findings thus warrant further dimensional investigation of correlates. It would be beneficial for future studies to replicate these findings in clinical samples with official diagnoses, including disorders other than MDD, as our sample size did not allow for additional analyses based on specific diagnoses.

Interestingly, no significant group differences were observed in the DARS subscales related to consumption (food/drinks) and sensory experiences, despite higher overall symptomatology in the clinical group. One possible explanation is that these domains may be less sensitive to variations in psychiatric symptomatology in undiagnosed or mixed samples, particularly when symptoms are below the clinical threshold. It is also conceivable that basic sensory and consummatory pleasures, such as eating or enjoying smells and textures, may be less impaired than motivational or social aspects in individuals without formal diagnoses. Although our sample is limited to a younger university student population, it is possible that age-related decline in sensory perception (e.g., hearing [65] or tasting [66]) may affect different dimensions of anhedonia in more diverse populations. For example, one study found that self-reported smell loss and taste loss individually predicted meeting full PHQ-9 criteria for major depressive disorder [67], while another study identified a direct effect of functional hearing impairment on mood [68]. This may represent a confounding factor in future studies examining age-heterogeneous samples. The DARS does not explicitly distinguish between anticipatory and consummatory components of anhedonia; however, prior research suggests that anticipatory impairments often emerge earlier and are more strongly linked to motivational deficits, while consummatory pleasures may remain relatively preserved in non-clinical or mildly affected individuals [4]. This could partly explain why group differences were more evident in social and activity domains, which may be more reliant on motivational engagement. Future studies should examine whether these patterns persist in clinical samples with confirmed diagnoses and explore whether specific domains of anhedonia follow different trajectories across psychiatric conditions.

It is noteworthy that the present study revealed stronger anhedonia symptomatology among male participants compared to their female counterparts. Previous studies have yielded conflicting results, with a limited number of studies conducted to date. These inconsistencies may be partly due to methodological differences, particularly regarding the types of instruments used and sample characteristics. Wellan et al. (2021), who also used the 17-item DARS in a healthy young adult sample, reported slightly higher anhedonia scores in women compared to men, suggesting that gender differences may emerge subtly and vary depending on sample characteristics, cultural context, or the presence of subclinical symptoms [17]. Another study identified men in a group of patients with subthreshold depression as displaying stronger symptoms [69]. The DARS utilizes individualized, preference-based items that are designed to mitigate the impact of gender, cultural, and subjective variability. In contrast, some questionnaires utilize general items that lack individualization. Consequently, the observed gender difference in the findings might be indicative of genuine disparities in hedonic processing in the student population as opposed to measurement bias. It is also plausible that sample-specific characteristics or contextual variables, such as academic stress or gender-related coping styles in university students, as well as the relatively low number of male participants contributed to this outcome. In light of the current evidence on gender differences in anhedonia, further research is necessary to examine these patterns in both clinical and non-clinical populations using standardized, preference-based tools like the DARS.

### Negative symptoms as a strong predictor of anhedonia

The present study employed regression analysis to explore the predictive values of various psychological factors on anhedonia. A significant regression equation was found, indicating that the model including anxiety, depression, negative symptoms, and the ISR for total symptom burden explained approximately 25.9% of the variance in anhedonia scores (model 1). This level of explanatory power suggests that, while the model captures meaningful relationships, there are likely other, unmeasured factors contributing to anhedonia that warrant further investigation. A second exploratory regression analysis (model 2) was conducted using all scales as predictors to identify additional differentiations between symptom burdens assessed by the ISR and determine the predictive value of stress. This model 2 explained 24.4% of the variance in anhedonia scores.

Negative symptoms were identified as a significant predictor of anhedonia in both the full sample and the clinical group, supporting the view of anhedonia as a core negative symptom [70] and its occurrence in diverse psychiatric disorders [71]. Negative symptoms, including diminishment of positive affect, social withdrawal, limited speech (alogia), and a lack of motivation (avolition), are key characteristics of various psychiatric disorders, like schizophrenia [72,73] and schizoaffective disorders [74]. Previous research identified the five-factor structure of negative symptoms, wherein the factors anhedonia, asociality, avolition, blunted affect, and alogia (lack of and poverty of speech) are correlated [75]. Negative symptoms show an intersection with depressive symptoms, as both are characterized by blunted affect, anhedonia, and reduced expression [76,77]. The strong association between negative symptoms and anhedonia observed in the present study may be explained by shared neurobiological underpinnings. Anhedonia and related negative symptoms have been linked to impairments in the mesocorticolimbic dopamine pathway, which encompasses key regions such as the ventral tegmental area, nucleus accumbens, and prefrontal cortex [78,79]. Abnormalities in dopaminergic signaling within these regions are thought to disrupt reward anticipation, motivation, and reinforcement learning processes central to both anhedonia and avolition. Neuroimaging studies have demonstrated reduced activation in these regions during reward processing tasks in individuals with schizophrenia and other disorders characterized by prominent negative symptoms [80,81]. Furthermore, glutamatergic dysregulation has been implicated in disrupting frontostriatal connectivity, which contributes to diminished affective and motivational responses [82]. The collective findings suggest that the association between negative symptoms and anhedonia is not only behavioral but also rooted in consistent alterations in dopaminergic and limbic-prefrontal circuits. Future studies should further explore these mechanisms in both clinical and non-clinical populations, ideally combining behavioral and neuroimaging approaches to disentangle the specific pathways involved.

Interestingly, depression scores did not predict anhedonia in the regression model. Previous studies found anhedonia to correlate with depression scores in MDD patients [83–85]. Exploratory results highlight the role of depression, as measured by the DASS, as a significant predictor when examined in isolation. However, its significance decreased when other predictors were included in the model. This suggests that while depression is relevant, its predictive power may be influenced by other related factors, such as anxiety or stress, which often co-occur. The reduced effect may also indicate shared variance between the predictors, making it difficult to isolate the unique contribution of depression. Thus, depression may not act independently, highlighting the importance of considering multiple factors in psychological assessments. In summary, while depression was initially a strong predictor, its diminished significance in the full model suggests that its association may be overlapping with or overshadowed by other variables. This highlights the importance of considering a multifaceted approach when examining psychological outcomes, as focusing on a single predictor may not capture the full complexity of these interrelated emotional and psychological processes. Further research could explore the relationships between these variables in more depth and larger samples that provide enough power for incorporating more variables in the models, particularly examining the role of potential mediators or moderators that may influence the predictive ability of depression in more complex models.

### Anxiety’s role in anhedonia in clinical populations

An exploratory analysis conducted on the clinical group alone yielded additional insights regarding the predictive value of different psychiatric symptoms for anhedonia. The adjusted regression model demonstrated a greater capacity of 33.21% to explain the variance in anhedonia compared to the full sample, suggesting that these predictors may be more relevant or stronger in individuals with clinical symptoms. This finding is consistent with the idea that psychological symptoms and their relationships with anhedonia may manifest more clearly in clinical populations where symptoms are more pronounced. Anxiety, as measured by the DASS, demonstrated a trend towards significance in the full sample, indicating that anxiety may also be a contributing factor in anhedonia, albeit to a lesser extent compared to negative symptoms. Notably, anxiety emerged as a significant predictor in the clinically noticeable group. Anxiety’s significant contribution to anhedonia in this group could be linked to the heightened state of arousal and fear typically associated with anxiety disorders, which may further disrupt reward processing and reduce the individuals’ ability and motivation to derive pleasure from typically enjoyable activities [33,34]. Considering the model proposed by Winer et al. (2018), which suggests that anhedonia acts as a mediator for the relationship between anxiety and depression rather than the other way around [33,34], our findings support this model given the predictive value of anxiety symptoms for anhedonia. In conclusion, anxiety represents a significant factor in the context of anhedonia in clinical populations. Further research is required to elucidate the underlying mechanisms, including moderation and mediation. Such insights could prove invaluable in identifying efficacious therapeutic interventions for anhedonia, given its association with poorer treatment outcomes and treatment resistance in depression.

Interestingly, the marginal significance of the ISR OCD scores observed in the full sample was not replicated in the clinical group. This divergence might suggest that obsessive-compulsive tendencies are less influential in clinical populations with more severe psychopathology, or that the relationship between OCD symptoms and anhedonia is more nuanced and influenced by additional factors not captured in the current model. Future research should explore this dynamic further, as OCD-related symptoms could affect anhedonia in more specific contexts or subpopulations. In a study by Abramovich et al. (2014), the prevalence of anhedonia in patients diagnosed with OCD was found to be 28.3% [86]. As with the full-sample analysis, other predictors, including depression, stress (DASS), and several ISR scales (including depression, OCD, somatoform, and eating disorder scales), did not significantly predict anhedonia in the regression model for the clinical group.

### Better fit of the 17-item DARS version

The general factor analysis of the 26-item version indicated a moderate model fit for the four-factor structure. In accordance with conventional fit index thresholds, a CFI below .90 and a TLI below .90 indicate that the model does not provide an optimal fit to the data [87]. Similarly, the RMSEA value of .082, which exceeds the recommended upper threshold of .08 [88], provides further support for this interpretation, indicating a marginal fit. Nevertheless, the SRMR value of .074 is within the acceptable range, as values below .08 typically indicate a reasonable fit [87]. In contrast, the analysis of the 17-item version yielded superior fit for the four-factor structure when compared to the 26-item model. In particular, the CFI value exceeds the .90 threshold, indicating a good fit. Although the TLI (TLI = .894) is slightly below the ideal threshold of .90, it nevertheless indicates a notable enhancement in fit relative to the 26-item version. Furthermore, the RMSEA value of .067 is considerably below the .08 threshold, indicating a superior alignment with the model. The SRMR value of .072 remains within the acceptable range. The 17-item model demonstrates an enhanced fit as evidenced by the lower AIC (4308.1) and BIC (4422.2) values in comparison to the 26-item version (AIC = 6391, BIC = 6556.8). Lower AIC and BIC values indicate that the 17-item model exhibits a superior balance between model fit and parsimony, thereby affirming its relative superiority over the 26-item version. Wellan et al. (2021) performed a multigroup CFA for the German 17-item version of the DARS and yielded slightly better CFI and TLI values in a healthy sample of young adults (mean age 24.4 years, CFI = .933, TLI = .920, RMSEA = .061 [90% CI = .053, .069], SRMR = .054, χ^2^_(113)_ = 306.04). This could be due to the usage of Maximum Likelihood Robust (MLR) method, which could not be applied in our analysis due to insufficient sample size. As stated by Li (2015), the MLR method can be applied with samples comprising over 200 participants [89].

Both findings suggest that the 17-item version may be a more efficient and accurate tool for measuring anhedonia in clinical and research contexts. Consequently, its implementation in clinical settings offers several advantages. The reduced length enhances its feasibility in time-constrained environments, such as outpatient psychiatric clinics, while maintaining the multidimensional assessment of anhedonia. The scale’s four domains (hobbies, food/drink, social activities, and sensory experiences) allow clinicians to identify specific areas of hedonic impairment, which can support individualized treatment planning. The demonstrated model fit in non-clinical samples, along with the ability to use the scale in smaller samples, also suggests its suitability for routine screening and longitudinal monitoring, including in early intervention or low-resource contexts.

Our findings demonstrate that the 17-item version can be applied in smaller samples with a reasonably good fit. For research purposes, the 17-item version facilitates its integration into broader assessment batteries and makes it well-suited for studies with repeated measurements. Its enhanced structural consistency supports further cross-cultural validation and its application across diverse clinical populations where anhedonia plays a central role, including major depressive disorder, schizophrenia, and PTSD.

In summary, the 17-item DARS demonstrates a pragmatic and psychometrically sound instrument for clinical applications and future investigations into the dimensional structure of anhedonia. The 26-item version of the DARS does not strongly adhere to the proposed four-factor structure of the 17-item version and requires further validation.

## Limitations

Although self-report scales such as the MAP-SR, DASS, and ISR have been extensively validated, they may be vulnerable to response biases, including social desirability and challenges in accurately self-assessing symptoms. Regarding the conceptualization of anhedonia, the DARS does not permit differentiation between the common subtypes of anticipatory and consummatory anhedonia. Consequently, the study was unable to investigate the distinctiveness of these subtypes in the student sample.

Furthermore, the recruitment strategy, which primarily targeted university students, may have introduced a potential selection bias, as this population may not be representative of the general population or clinical populations. Typically, university students are younger, more educated, and may differ in terms of stressors, lifestyle, and mental health experiences compared to non-student or clinical samples. This homogeneity could have influenced the variability and generalizability of the findings, particularly regarding the expression and severity of anhedonic symptoms [90]. To enhance the external validity of future research, it is recommended to include more diverse samples, including individuals from clinical settings.

Additionally, the formation of groups based on screening questionnaires and cut-off values may have introduced bias, particularly in the assessment of symptoms. It is imperative to consider the constraints of the 17-item version of the Dimensional Anhedonia Rating Scale (DARS). The DARS offers a multifaceted evaluation of anhedonia, encompassing various domains. However, it falls short of differentiating between the anticipatory and consummatory components of anhedonia. These two subtypes are distinguished by their distinct neurobiological and clinical implications. While the DARS demonstrates adequate internal consistency and convergent validity, its relatively brief format and reliance on self-report may reduce sensitivity to subtle individual differences. These limitations may have influenced the present study’s ability to detect nuanced symptom patterns and should be considered when interpreting the findings. Only a small number of subjects self-reported a diagnosis, which made it challenging to compare the results with those of previous studies on clinical samples. Participants were included if they took psychopharmacological medication. Despite the fact that neither medication nor treatment significantly predicted anhedonia, these parameters are still worth considering when analyzing symptomatology. Incorporating clinician-based grouping or sampling from patients could enhance the robustness of future research endeavors. The present study is constrained by the relatively modest sample size, which precluded the application of the MLR method. Due to the small size of some subgroups, particularly those involving participants on medication, analyses may be underpowered to detect smaller effects. Findings, especially of exploratory analyses, should thus be interpreted with caution, and replication in larger samples is warranted. Additionally, the present study’s sample included a rather small number of male participants, which may have influenced the representativeness of the findings with respect to gender.

## Conclusion

In conclusion, this study highlights the complexity of anhedonia, revealing that while negative symptoms are a significant predictor, the relationship between anhedonia and other psychological factors, such as anxiety and depression, is more intricate than initially anticipated. The regression analyses suggest that anxiety and negative symptoms play a stronger role in clinical populations, with anxiety emerging as a key predictor in individuals meeting cut-off scores. However, depression’s predictive power diminished when considered alongside other variables, indicating shared variance with anxiety and stress. Factor analyses suggested that the 17-item version of the DARS provides a superior fit than the 26-item version, supporting its use in both clinical and research settings. Overall, the findings underscore the need for further research on anhedonia in clinical samples to refine current dimensional models and better understand psychological and neurocognitive underpinnings.

## Supporting information

S1 Table Individual preferences participants provided for the DARS. Participants were asked to provide a list of two to three individual preferences to be considered while completing the Dimensional Anhedonia Rating Scale (DARS). The following questions were posed: Activity “Please list at least 2 of your favorite pastimes/hobbies that are NOT primarily social”; Consumption “Please list at least 2 of your favorite foods/drinks”; Experiences “Please list at least 2 of your favorite sensory experiences”; Social “Please list at least 2 of your favorite social activities”.
(DOCX)

S2 Fig Correlation matrix. (DOCX)

S3 Table Results from multiple linear regression analysis including gender. (DOCX)

S4 Table Results from multiple linear regression analysis for the clinical group. (DOCX)

S5 Table Results from multiple linear regression analysis for the clinical group including psychiatric factors. (DOCX)

## References

[1] Buckner JD, Joiner TE Jr, Pettit JW, Lewinsohn PM, Schmidt NB. Implications of the DSM’s emphasis on sadness and anhedonia in major depressive disorder. Psychiatry Res. 2008;159(1–2):25–30. doi: 10.1016/j.psychres.2007.05.010 18334272 PMC3688280

[2] Lambert C, Da Silva S, Ceniti AK, Rizvi SJ, Foussias G, Kennedy SH. Anhedonia in depression and schizophrenia: A transdiagnostic challenge. CNS Neurosci Ther. 2018;24(7):615–23. doi: 10.1111/cns.12854 29687627 PMC6489811

[3] Watson R, Harvey K, McCabe C, Reynolds S. Understanding anhedonia: a qualitative study exploring loss of interest and pleasure in adolescent depression. Eur Child Adolesc Psychiatry. 2019;29(4):489–99. doi: 10.1007/s00787-019-01364-y 31270605 PMC7103575

[4] Gard DE, Kring AM, Gard MG, Horan WP, Green MF. Anhedonia in schizophrenia: distinctions between anticipatory and consummatory pleasure. Schizophr Res. 2007;93(1–3):253–60. doi: 10.1016/j.schres.2007.03.008 17490858 PMC1986826

[5] Nawijn L, van Zuiden M, Frijling JL, Koch SBJ, Veltman DJ, Olff M. Reward functioning in PTSD: a systematic review exploring the mechanisms underlying anhedonia. Neurosci Biobehav Rev. 2015;51:189–204. doi: 10.1016/j.neubiorev.2015.01.019 25639225

[6] Garfield JBB, Lubman DI, Yücel M. Anhedonia in substance use disorders: a systematic review of its nature, course and clinical correlates. Aust N Z J Psychiatry. 2014;48(1):36–51. doi: 10.1177/0004867413508455 24270310

[7] Assogna F, Cravello L, Caltagirone C, Spalletta G. Anhedonia in Parkinson’s disease: a systematic review of the literature. Mov Disord. 2011;26(10):1825–34. doi: 10.1002/mds.23815 21661052

[8] Ribot T. La psychologie des sentiment (The psychology of feelings). Paris: Felix Alcan; 1896.

[9] Gao K, Sweet J, Su M, Calabrese JR. Depression severity and quality of life of qualified and unqualified patients with a mood disorder for a research study targeting anhedonia in a clinical sample. Asian Journal of Psychiatry. 2017;27:40–7. doi: 10.1016/j.ajp.2017.02.013 28558894

[10] Ritsner MS, Arbitman M, Lisker A. Anhedonia is an important factor of health-related quality-of-life deficit in schizophrenia and schizoaffective disorder. J Nerv Ment Dis. 2011;199(11):845–53. doi: 10.1097/NMD.0b013e3182349ce6 22048136

[11] Ducasse D, Loas G, Dassa D, Gramaglia C, Zeppegno P, Guillaume S, et al. Anhedonia is associated with suicidal ideation independently of depression: A meta-analysis. Depress Anxiety. 2018;35(5):382–92. doi: 10.1002/da.22709 29232491

[12] Ely BA, Nguyen TNB, Tobe RH, Walker AM, Gabbay V. Multimodal Investigations of Reward Circuitry and Anhedonia in Adolescent Depression. Front Psychiatry. 2021;12:678709. doi: 10.3389/fpsyt.2021.678709 34366915 PMC8345280

[13] Guineau MG, Ikani N, Rinck M, Collard RM, van Eijndhoven P, Tendolkar I, et al. Anhedonia as a transdiagnostic symptom across psychological disorders: a network approach. Psychol Med. 2023;53(9):3908–19. doi: 10.1017/S0033291722000575 35348051 PMC10317820

[14] Chan RCK, Wang Y, Yan C, Zhao Q, McGrath J, Hsi X, et al. A study of trait anhedonia in non-clinical Chinese samples: evidence from the Chapman Scales for Physical and Social Anhedonia. PLoS One. 2012;7(4):e34275. doi: 10.1371/journal.pone.0034275 22529910 PMC3328477

[15] Dodell-Feder D, Germine L. Epidemiological Dimensions of Social Anhedonia. Clinical Psychological Science. 2018;6(5):735–43. doi: 10.1177/2167702618773740

[16] Martino I, Santangelo G, Moschella D, Marino L, Servidio R, Augimeri A, et al. Assessment of Snaith-Hamilton Pleasure Scale (SHAPS): the dimension of anhedonia in Italian healthy sample. Neurol Sci. 2018;39(4):657–61. doi: 10.1007/s10072-018-3260-2 29383616

[17] Wellan SA, Daniels A, Walter H. State Anhedonia in Young Healthy Adults: Psychometric Properties of the German Dimensional Anhedonia Rating Scale (DARS) and Effects of the COVID-19 Pandemic. Front Psychol. 2021;12:682824. doi: 10.3389/fpsyg.2021.682824 34248783 PMC8260844

[18] Luby JL, Agrawal A, Belden A, Whalen D, Tillman R, Barch DM. Developmental Trajectories of the Orbitofrontal Cortex and Anhedonia in Middle Childhood and Risk for Substance Use in Adolescence in a Longitudinal Sample of Depressed and Healthy Preschoolers. Am J Psychiatry. 2018;175(10):1010–21. doi: 10.1176/appi.ajp.2018.17070777 29558817 PMC6150861

[19] Stringaris A, Vidal-Ribas Belil P, Artiges E, Lemaitre H, Gollier-Briant F, Wolke S, et al. The Brain’s Response to Reward Anticipation and Depression in Adolescence: Dimensionality, Specificity, and Longitudinal Predictions in a Community-Based Sample. Am J Psychiatry. 2015;172(12):1215–23. doi: 10.1176/appi.ajp.2015.14101298 26085042 PMC7614275

[20] Cuthbert BN. The RDoC framework: facilitating transition from ICD/DSM to dimensional approaches that integrate neuroscience and psychopathology. World Psychiatry. 2014;13(1):28–35. doi: 10.1002/wps.20087 24497240 PMC3918011

[21] Treadway MT, Zald DH. Reconsidering anhedonia in depression: lessons from translational neuroscience. Neurosci Biobehav Rev. 2011;35(3):537–55. doi: 10.1016/j.neubiorev.2010.06.006 20603146 PMC3005986

[22] Der-Avakian A, Markou A. The neurobiology of anhedonia and other reward-related deficits. Trends Neurosci. 2012;35(1):68–77. doi: 10.1016/j.tins.2011.11.005 22177980 PMC3253139

[23] Rizvi SJ, Quilty LC, Sproule BA, Cyriac A, Michael Bagby R, Kennedy SH. Development and validation of the Dimensional Anhedonia Rating Scale (DARS) in a community sample and individuals with major depression. Psychiatry Res. 2015;229(1–2):109–19. doi: 10.1016/j.psychres.2015.07.062 26250147

[24] Chapman LJ, Chapman JP, Raulin ML. Scales for physical and social anhedonia. J Abnorm Psychol. 1976;85(4):374–82. doi: 10.1037//0021-843x.85.4.374 956504

[25] Eckblad ML, Chapman LJ, Chapman JP. The revised social anhedonia scale. 1982.

[26] Gard DE, Gard MG, Kring AM, John OP. Anticipatory and consummatory components of the experience of pleasure: A scale development study. Journal of Research in Personality. 2006;40(6):1086–102. doi: 10.1016/j.jrp.2005.11.001

[27] Klein D. Depression and Anhedonia. Anhedonia and Affective States. New York: PMA Publishing. 1984. p. 1–14.

[28] Snaith RP, Hamilton M, Morley S, Humayan A, Hargreaves D, Trigwell P. A scale for the assessment of hedonic tone the Snaith-Hamilton Pleasure Scale. Br J Psychiatry. 1995;167(1):99–103. doi: 10.1192/bjp.167.1.99 7551619

[29] American Psychiatric Association, editor. Diagnostic and Statistical Manual of Mental Disorders. Fifth Edition. 2013. Available: https://psychiatryonline.org/doi/book/10.1176/appi.books.9780890425596

[30] Treadway MT, Zald DH. Reconsidering anhedonia in depression: Lessons from translational neuroscience. Neuroscience & Biobehavioral Reviews. 2011;35(3):537–55. doi: 10.1016/j.neubiorev.2010.06.006 20603146 PMC3005986

[31] Pizzagalli DA. Depression, stress, and anhedonia: toward a synthesis and integrated model. Annu Rev Clin Psychol. 2014;10:393–423. doi: 10.1146/annurev-clinpsy-050212-185606 24471371 PMC3972338

[32] Trøstheim M, Eikemo M, Meir R, Hansen I, Paul E, Kroll SL, et al. Assessment of Anhedonia in Adults With and Without Mental Illness: A Systematic Review and Meta-analysis. JAMA Netw Open. 2020;3(8):e2013233. doi: 10.1001/jamanetworkopen.2020.13233 32789515 PMC7116156

[33] Winer ES, Bryant J, Bartoszek G, Rojas E, Nadorff MR, Kilgore J. Mapping the relationship between anxiety, anhedonia, and depression. J Affect Disord. 2017;221:289–96. doi: 10.1016/j.jad.2017.06.006 28668590 PMC6080718

[34] Calafiore C, Collins AC, Bartoszek G, Winer ES. Assessing relinquishment of positivity as a central symptom bridging anxiety and depression. Journal of Affective Disorders. 2024;367:38–48. doi: 10.1016/j.jad.2024.08.031 39147161 PMC11496000

[35] Slavich GM, Irwin MR. From stress to inflammation and major depressive disorder: a social signal transduction theory of depression. Psychol Bull. 2014;140(3):774–815. doi: 10.1037/a0035302 24417575 PMC4006295

[36] Bogdan R, Pizzagalli DA. Acute stress reduces reward responsiveness: implications for depression. Biol Psychiatry. 2006;60(10):1147–54. doi: 10.1016/j.biopsych.2006.03.037 16806107 PMC2288705

[37] Berghorst LH, Bogdan R, Frank MJ, Pizzagalli DA. Acute stress selectively reduces reward sensitivity. Front Hum Neurosci. 2013;7:133. doi: 10.3389/fnhum.2013.00133 23596406 PMC3622896

[38] Robotham D, Julian C. Stress and the higher education student: a critical review of the literature. Journal of Further and Higher Education. 2006;30(2):107–17. doi: 10.1080/03098770600617513

[39] Auerbach RP, Mortier P, Bruffaerts R, Alonso J, Benjet C, Cuijpers P, et al. WHO World Mental Health Surveys International College Student Project: Prevalence and distribution of mental disorders. J Abnorm Psychol. 2018;127(7):623–38. doi: 10.1037/abn0000362 30211576 PMC6193834

[40] Adewuya AO, Coker OA, Atilola O, Ola BA, Zachariah MP, Adewumi T, et al. Gender difference in the point prevalence, symptoms, comorbidity, and correlates of depression: findings from the Lagos State Mental Health Survey (LSMHS), Nigeria. Arch Womens Ment Health. 2018;21(6):591–9. doi: 10.1007/s00737-018-0839-9 29594370

[41] Fonseca-Pedrero E, Lemos-Giráldez S, Muñiz J, García-Cueto E, Campillo-Alvarez A. Schizotypy in adolescence: the role of gender and age. J Nerv Ment Dis. 2008;196(2):161–5. doi: 10.1097/NMD.0b013e318162aa79 18277226

[42] Saucier G, Kenner J, Iurino K, Bou Malham P, Chen Z, Thalmayer AG, et al. Cross-Cultural Differences in a Global “Survey of World Views”. Journal of Cross-Cultural Psychology. 2014;46(1):53–70. doi: 10.1177/0022022114551791

[43] Jager J, Putnick DL, Bornstein MH. II. More Than Just Convenient: The Scientific Merits Of Homogeneous Convenience Samples. Monogr Soc Res Child Dev. 2017;82(2):13–30. doi: 10.1111/mono.12296 28475254 PMC5606225

[44] Ibrahim AK, Kelly SJ, Adams CE, Glazebrook C. A systematic review of studies of depression prevalence in university students. J Psychiatr Res. 2013;47(3):391–400. doi: 10.1016/j.jpsychires.2012.11.015 23260171

[45] APA dictionary of psychology. Washington, DC, US: American Psychological Association. 2007.

[46] Engel M, Lincoln TM. MAP-SR - Motivation and Pleasure Scale - Self-Report - deutsche Fassung. 2017 [cited 14 Apr 2025]. Available: https://www.psycharchives.org/en/item/e4452965-f550-4707-9287-270990c93d22

[47] Nilges P, Essau C. DASS. Depressions-Angst-Stress-Skalen - deutschsprachige Kurzfassung. ZPID (Leibniz Institute for Psychology) – Open Test Archive; 2021. doi: 10.23668/PSYCHARCHIVES.4579

[48] Tritt K, von Heymann F, Zaudig M, Zacharias I, Söllner W, Loew T. Development of the “ICD-10-Symptom-Rating”(ISR) questionnaire. Z Psychosom Med Psychother. 2008;54(4):409–18. doi: 10.13109/zptm.2008.54.4.409 19049690

[49] Blackwell SE, Westermann K, Woud ML, Cwik JC, Neher T, Graz C, et al. Computerized positive mental imagery training versus cognitive control training versus treatment as usual in inpatient mental health settings: study protocol for a randomized controlled feasibility trial. Pilot Feasibility Stud. 2018;4:133. doi: 10.1186/s40814-018-0325-1 30123524 PMC6091085

[50] Engel M, Lincoln TM. Motivation and Pleasure Scale-Self-Report (MAP-SR): Validation of the German version of a self-report measure for screening negative symptoms in schizophrenia. Compr Psychiatry. 2016;65:110–5. doi: 10.1016/j.comppsych.2015.11.001 26773998

[51] Nilges P, Essau C. Die Depressions-Angst-Stress-Skalen. Schmerz. 2015;29: 649–57. doi: 10.1007/s00482-015-0019-z 26205682

[52] Braun SR, Zimmermann J, Söhner S, Neidhardt S. Validity of the ICD-10 Symptom Rating (ISR) in a Non-Clinical Sample. Psychother Psychosom Med Psychol. 2017;67(11):477–84. doi: 10.1055/s-0043-118654 29121683

[53] Hoheisel M, Popkirov S, Michaelis R, Rose M. Psychobehavioral B-Criteria of Somatic Symptom Disorder Are Associated with Somatic Symptom Reporting in a Large Sample of Psychosomatic Outpatients. Complex Psychiatry. 2024;10(1–4):10–8. doi: 10.1159/000536668 38455259 PMC10917430

[54] Robin X, Turck N, Hainard A, Tiberti N, Lisacek F, Sanchez J-C, et al. pROC: an open-source package for R and S+ to analyze and compare ROC curves. BMC Bioinformatics. 2011;12:77. doi: 10.1186/1471-2105-12-77 21414208 PMC3068975

[55] Nahm FS. Receiver operating characteristic curve: overview and practical use for clinicians. Korean J Anesthesiol. 2022;75(1):25–36. doi: 10.4097/kja.21209 35124947 PMC8831439

[56] Cook RD. Detection of influential observations in linear regression. Technometrics. 1977;19:I5–8.

[57] Faul F, Erdfelder E, Buchner A, Lang A-G. Statistical power analyses using G*Power 3.1: tests for correlation and regression analyses. Behav Res Methods. 2009;41(4):1149–60. doi: 10.3758/BRM.41.4.1149 19897823

[58] R Core Team. R: A language and environment for statistical computing. R Foundation for Statistical Computing, Vienna, Austria. 2021. Available: https://www.R-project.org/

[59] Posit team. RStudio: Integrated Development Environment for R. PBC, Boston, MA: Posit Software; 2024. Available: http://www.posit.co/

[60] Zeileis A, Hothorn T. Diagnostic checking in regression relationships. R News. 2002;2:7–1.

[61] Fox J, Weisberg S. An R Companion to Applied Regression. third ed. Thousand Oaks CA: Sage. 2019.

[62] Rosseel Y. lavaan: AnRPackage for Structural Equation Modeling. J Stat Soft. 2012;48(2). doi: 10.18637/jss.v048.i02

[63] Gorostowicz A, Rizvi SJ, Kennedy SH, Chrobak AA, Dudek D, Cyranka K, et al. Polish adaptation of the Dimensional Anhedonia Rating Scale (DARS) - validation in the clinical sample. Front Psychiatry. 2023;14:1268290. doi: 10.3389/fpsyt.2023.1268290 37817828 PMC10561247

[64] Lin J, Su Y, Rizvi SJ, Jagoda J, Li J, Wu Y, et al. Define and characterize the anhedonia in major depressive disorder: An explorative study. J Affect Disord. 2022;313:235–42. doi: 10.1016/j.jad.2022.06.082 35788366

[65] Dillard LK, Matthews LJ, Maldonado L, Simpson AN, Dubno JR. Demographic factors impact the rate of hearing decline across the adult lifespan. Commun Med (Lond). 2024;4(1):171. doi: 10.1038/s43856-024-00593-w 39215139 PMC11364848

[66] Alves LSM, Munduri JM de S, Mattos MC de O, Stefani CM, Dame-Teixeira N. Changes in taste perception in elderly population and its potential impact on oral health: a systematic review with meta-analysis. Front Oral Health. 2024;5:1517913. doi: 10.3389/froh.2024.1517913 39703868 PMC11655467

[67] Hur K, Choi JS, Zheng M, Shen J, Wrobel B. Association of alterations in smell and taste with depression in older adults. Laryngoscope Investig Otolaryngol. 2018;3(2):94–9. doi: 10.1002/lio2.142 29721540 PMC5915822

[68] Brink P, Stones M. Examination of the relationship among hearing impairment, linguistic communication, mood, and social engagement of residents in complex continuing-care facilities. Gerontologist. 2007;47(5):633–41. doi: 10.1093/geront/47.5.633 17989405

[69] Crockett MA, Martínez V, Jiménez-Molina Á. Subthreshold depression in adolescence: Gender differences in prevalence, clinical features, and associated factors. J Affect Disord. 2020;272:269–76. doi: 10.1016/j.jad.2020.03.111 32553367

[70] Blanchard JJ, Cohen AS. The structure of negative symptoms within schizophrenia: implications for assessment. Schizophr Bull. 2006;32(2):238–45. doi: 10.1093/schbul/sbj013 16254064 PMC2632211

[71] Jarratt-Barnham I, Saleh Y, Husain M, Kirkpatrick B, Fernandez-Egea E. The influence of negative and affective symptoms on anhedonia self-report in schizophrenia. Compr Psychiatry. 2020;98:152165. doi: 10.1016/j.comppsych.2020.152165 32007758

[72] Loas G, Noisette C, Legrand A, Boyer P. Is anhedonia a specific dimension in chronic schizophrenia?. Schizophr Bull. 2000;26(2):495–506. doi: 10.1093/oxfordjournals.schbul.a033468 10885646

[73] Correll CU, Schooler NR. Negative Symptoms in Schizophrenia: A Review and Clinical Guide for Recognition, Assessment, and Treatment. Neuropsychiatr Dis Treat. 2020;16:519–34. doi: 10.2147/NDT.S225643 32110026 PMC7041437

[74] Muñoz-Negro JE, Lozano V, Ibanez-Casas I, de la Cruz BG, Soler A, Alcalá JA, et al. Negative symptoms across psychotic spectrum disorders. The European Journal of Psychiatry. 2017;31(1):37–41. doi: 10.1016/j.ejpsy.2016.12.002

[75] Paul NB, Strauss GP, Gates-Woodyatt JJ, Barchard KA, Allen DN. Two and five-factor models of negative symptoms in schizophrenia are differentially associated with trait affect, defeatist performance beliefs, and psychosocial functioning. Eur Arch Psychiatry Clin Neurosci. 2023;273: 1715–24. doi: 10.1007/s00406-022-01507-4 36633673

[76] Fuentes-Claramonte P, Garcia-Leon MA, Salgado-Pineda P, Ramiro N, Soler-Vidal J, Torres ML, et al. Do the negative symptoms of schizophrenia reflect reduced responsiveness to reward? Examination using a reward prediction error (RPE) task. Psychol Med. 2023;53(15):7106–15. doi: 10.1017/S0033291723000521 36987680 PMC10719670

[77] Moran EK, Culbreth AJ, Barch DM. Anhedonia in Schizophrenia. In: Pizzagalli DA, editor. Anhedonia: Preclinical, Translational, and Clinical Integration. Cham: Springer International Publishing; 2022. pp. 129–145. doi:10.1007/7854_2022_321

[78] Millan MJ, Fone K, Steckler T, Horan WP. Negative symptoms of schizophrenia: clinical characteristics, pathophysiological substrates, experimental models and prospects for improved treatment. Eur Neuropsychopharmacol. 2014;24(5):645–92. doi: 10.1016/j.euroneuro.2014.03.008 24820238

[79] Zhang B, Lin P, Shi H, Öngür D, Auerbach RP, Wang X, et al. Mapping anhedonia-specific dysfunction in a transdiagnostic approach: an ALE meta-analysis. Brain Imaging Behav. 2016;10(3):920–39. doi: 10.1007/s11682-015-9457-6 26487590 PMC4838562

[80] Wang X, Zhang Y, Huang J, Wang Y, Niu Y, Lui SSY, et al. Revisiting reward impairments in schizophrenia spectrum disorders: a systematic review and meta-analysis for neuroimaging findings. Psychol Med. 2023;53(15):7189–202. doi: 10.1017/S0033291723000703 36994747

[81] Esslinger C, Englisch S, Inta D, Rausch F, Schirmbeck F, Mier D, et al. Ventral striatal activation during attribution of stimulus saliency and reward anticipation is correlated in unmedicated first episode schizophrenia patients. Schizophr Res. 2012;140(1–3):114–21. doi: 10.1016/j.schres.2012.06.025 22784688

[82] Yan Z, Rein B. Mechanisms of synaptic transmission dysregulation in the prefrontal cortex: pathophysiological implications. Mol Psychiatry. 2022;27(1):445–65. doi: 10.1038/s41380-021-01092-3 33875802 PMC8523584

[83] Gong L, Yin Y, He C, Ye Q, Bai F, Yuan Y, et al. Disrupted reward circuits is associated with cognitive deficits and depression severity in major depressive disorder. J Psychiatr Res. 2017;84:9–17. doi: 10.1016/j.jpsychires.2016.09.016 27673704

[84] Pelizza L, Ferrari A. Anhedonia in schizophrenia and major depression: state or trait?. Ann Gen Psychiatry. 2009;8:22. doi: 10.1186/1744-859X-8-22 19811665 PMC2764701

[85] Pizzagalli DA, Holmes AJ, Dillon DG, Goetz EL, Birk JL, Bogdan R, et al. Reduced caudate and nucleus accumbens response to rewards in unmedicated individuals with major depressive disorder. Am J Psychiatry. 2009;166(6):702–10. doi: 10.1176/appi.ajp.2008.08081201 19411368 PMC2735451

[86] Abramovitch A, Pizzagalli DA, Reuman L, Wilhelm S. Anhedonia in obsessive-compulsive disorder: beyond comorbid depression. Psychiatry Res. 2014;216(2):223–9. doi: 10.1016/j.psychres.2014.02.002 24564999

[87] Byrne BM. Structural Equation Modelling with EQS and EQS/Windows: Basic Concepts, Applications, and Programming. Sage; 1994.

[88] MacCallum RC, Browne MW, Sugawara HM. Power analysis and determination of sample size for covariance structure modeling. Psychological Methods. 1996;1(2):130–49. doi: 10.1037/1082-989x.1.2.130

[89] Li C-H. Confirmatory factor analysis with ordinal data: Comparing robust maximum likelihood and diagonally weighted least squares. Behav Res Methods. 2016;48(3):936–49. doi: 10.3758/s13428-015-0619-7 26174714

[90] McCloud T, Kamenov S, Callender C, Lewis G, Lewis G. The association between higher education attendance and common mental health problems among young people in England: evidence from two population-based cohorts. The Lancet Public Health. 2023;8(10):e811–9. doi: 10.1016/s2468-2667(23)00188-3 PMC10958987 37777290

