# Supplementary material for: Happy or not? An investigative study on well-being and anhedonia in everyday life
Source: PLoS One. 2025 Sep 11;20(9):e0331769. doi: 10.1371/journal.pone.0331769 (PMC12425193; doi:10.1371/journal.pone.0331769)
Supplement: S1 Table — Participants were asked to provide a list of two to three individual preferences to be considered while completing the Dimensional Anhedonia Rating Scale (DARS). The following questions were posed: Activity “Please list at least 2 of your favorite pastimes/hobbies that are NOT primarily social”; Consumption “Please list at least 2 of your favorite foods/drinks”; Experiences “Please list at least 2 of your favorite sensory experiences”; Social “Please list at least 2 of your favorite social activities”. (DOCX) [file pone.0331769.s001.docx]

Supplementary Materials

Happy or not? An investigative study on Well-being and Anhedonia in Everyday Life

Merklein, Peterburs, Mundorf

**Table S1. Individual preferences participants provided for the DARS.** Participants were asked to provide a list of two to three individual preferences to be considered while completing the Dimensional Anhedonia Rating Scale (DARS). The following questions were posed: Activity “Please list at least 2 of your favorite pastimes/hobbies that are NOT primarily social”; Consumption “Please list at least 2 of your favorite foods/drinks”; Experiences “Please list at least 2 of your favorite sensory experiences”; Social “Please list at least 2 of your favorite social activities”.

| **activity** | **consumption** | **sensory experience** | **social** |
| --- | --- | --- | --- |
| Reading | Pizza | Listening to music | Meeting friends |
| Cooking | Sushi | Watching the sunset | Taking a walk |
| Doing sports | Coffee | Cuddling | Drinking coffee |
| Going for a walk | Pasta | Being in nature | Eating out |
| Dancing | Burgers | Hugging | Doing sports |
| Watching TV | Noodles | Travelling | Cooking together |
| Musical activities | Ice cream | Time with friends | Going out to party |
