# Supplementary material for: Happy or not? An investigative study on well-being and anhedonia in everyday life
Source: PLoS One. 2025 Sep 11;20(9):e0331769. doi: 10.1371/journal.pone.0331769 (PMC12425193; doi:10.1371/journal.pone.0331769)
Supplement: S2 Fig — (DOCX) [file pone.0331769.s002.docx]

Supplementary Materials

Happy or not? An investigative study on Well-being and Anhedonia in Everyday Life

Merklein, Peterburs, Mundorf

**Figure S2. Correlation matrix.** *DARS:* Dimensional Anhedonia Rating Scale; DASS: Depression Anxiety Stress Scales; MAP-SR: Motivation and Pleasure Scale - Self-Report; ISR: ICD-10-Symptom-Rating.

**
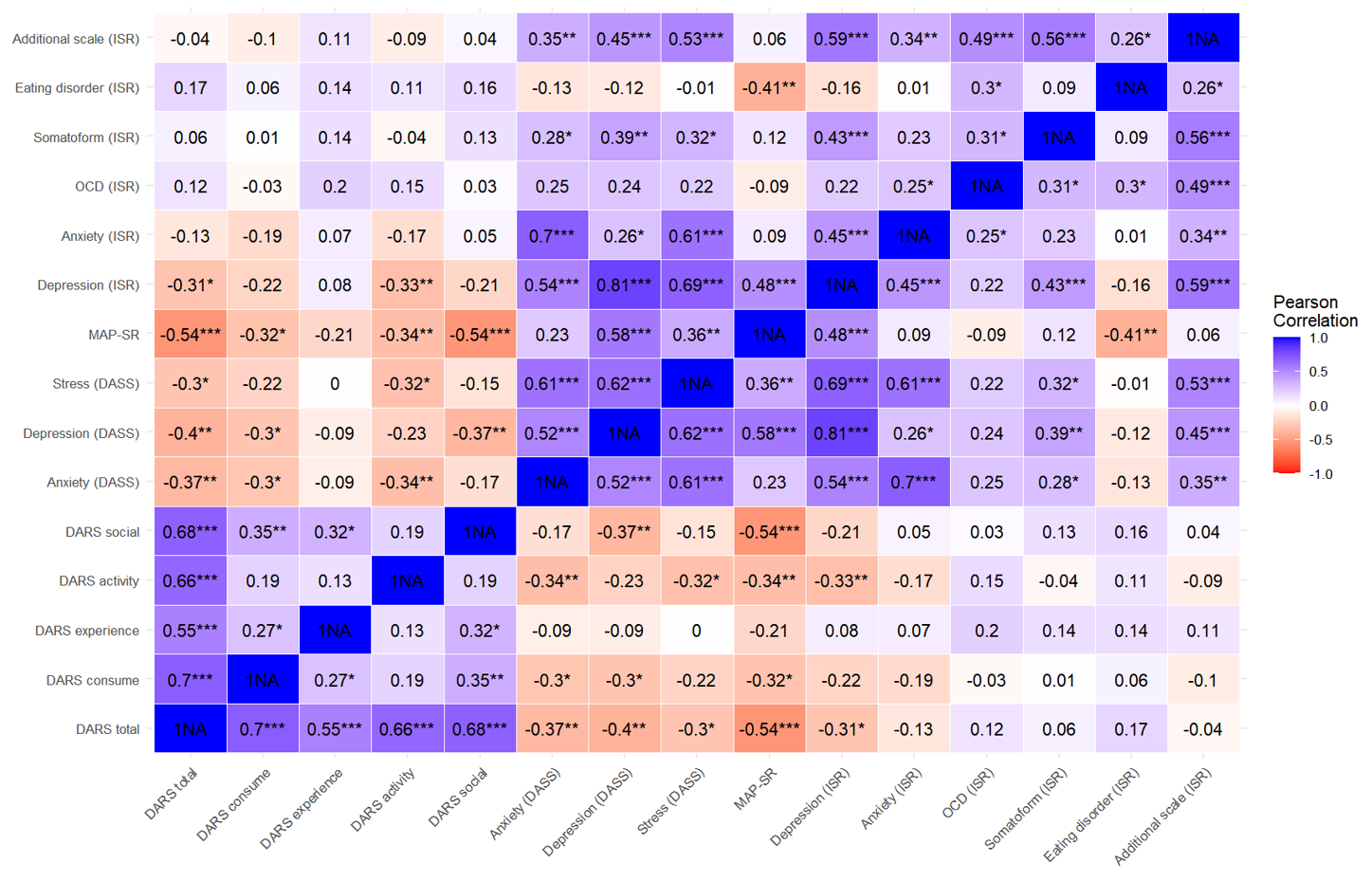
**
