# Supplementary material for: Happy or not? An investigative study on well-being and anhedonia in everyday life
Source: PLoS One. 2025 Sep 11;20(9):e0331769. doi: 10.1371/journal.pone.0331769 (PMC12425193; doi:10.1371/journal.pone.0331769)
Supplement: S3 Table — (DOCX) [file pone.0331769.s003.docx]

Supplementary Materials

Happy or not? An investigative study on Well-being and Anhedonia in Everyday Life

Merklein, Peterburs, Mundorf

**Table S3. Results from multiple linear regression analysis including gender.** *DARS:* Dimensional Anhedonia Rating Scale; DASS: Depression Anxiety Stress Scales; MAP-SR: Motivation and Pleasure Scale - Self-Report; ISR: ICD-10-Symptom-Rating; VIF: Variance Inflation Factor.

|  | Dependent variable: anhedonia (DARS) | | | | | |
| --- | --- | --- | --- | --- | --- | --- |
|  | Unstandardized coefficients | |  |  | *Collinearity* | |
| Predictor | *b* | *SE b* | **t** | ***p*** | *Tolerance* | *VIF* |
| (Intercept) | 93.32 | 2.19 | 42.57 | <.001*** |  |  |
| DASS anxiety | -0.60 | 0.32 | -1.91 | 0.059 | 0.269 | 3.720 |
| DASS depression | -0.36 | 0.28 | -1.29 | 0.199 | 0.233 | 4.299 |
| DASS stress | 0.24 | 0.23 | 1.03 | 0.306 | 0.393 | 2.542 |
| MAP-SR | -0.49 | 0.11 | -4.56 | <.001*** | 0.562 | 1.781 |
| ISR depression | 1.74 | 1.47 | 1.19 | 0.237 | 0.224 | 4.460 |
| ISR anxiety | 0.33 | 1.22 | 0.28 | 0.784 | 0.298 | 3.360 |
| ISR OCD | 2.10 | 0.96 | 2.20 | 0.030* | 0.618 | 1.619 |
| ISR somatoform | 0.89 | 1.09 | 0.82 | 0.414 | 0.600 | 1.668 |
| ISR eating disorder | -0.44 | 0.70 | -0.62 | 0.536 | 0.741 | 1.350 |
| ISR additional scale | -1.89 | 1.91 | -0.99 | 0.324 | 0.320 | 3.121 |
| Gender | 5.12 | 1.40 | 3.66 | <.001*** | 0.940 | 1.064 |
| *Multiple R*^2^  *Adjusted R*^2^ |  | 0.4126 0.3522 |  |  |  |  |
| *F* | 6.833 (11 and 107 DF) | | |  |  |  |
| *Residual SE* | 6.198 | | |  |  |  |
