# Supplementary material for: Happy or not? An investigative study on well-being and anhedonia in everyday life
Source: PLoS One. 2025 Sep 11;20(9):e0331769. doi: 10.1371/journal.pone.0331769 (PMC12425193; doi:10.1371/journal.pone.0331769)
Supplement: S4 Table — (DOCX) [file pone.0331769.s004.docx]

Supplementary Materials

Happy or not? An investigative study on Well-being and Anhedonia in Everyday Life

Merklein, Peterburs, Mundorf

**Table S4. Results from multiple linear regression analysis for the clinical group.** *DARS:* Dimensional Anhedonia Rating Scale; DASS: Depression Anxiety Stress Scales; MAP-SR: Motivation and Pleasure Scale - Self-Report; ISR: ICD-10-Symptom-Rating; VIF: Variance Inflation Factor.

|  | Dependent variable: anhedonia (DARS) | | | | | |
| --- | --- | --- | --- | --- | --- | --- |
|  | Unstandardized coefficients | |  |  | *Collinearity* | |
| Predictor | *b* | *SE b* | **t** | ***p*** | *Tolerance* | *VIF* |
| (Intercept) | 102.91 | 4.26 | 24.17 | < 2e-16 |  |  |
| DASS anxiety | -1.06 | .40 | -2.65 | .01* | .361 | 2.770 |
| DASS depression | -.03 | .42 | -.08 | .94 | .205 | 4.877 |
| DASS stress | -.04 | .37 | -.12 | .91 | .349 | 2.867 |
| MAP-SR | -.64 | .19 | -.44 | .001** | .501 | 1.995 |
| ISR depression | .08 | 2.23 | .04 | .97 | .200 | 4.990 |
| ISR anxiety | 1.73 | 1.64 | 1.06 | .29 | .347 | 2.885 |
| ISR OCD | 1.62 | 1.36 | 1.19 | .24 | .668 | 1.497 |
| ISR somatoform | 2.26 | 1.35 | 1.67 | .10 | .648 | 1.543 |
| ISR eating disorder | -1.13 | 1.00 | -1.14 | 2.6 | .634 | 1.578 |
| ISR additional scale | -.83 | 2.55 | -.32 | .75 | .359 | 2.785 |
| *Multiple R*^2^  *Adjusted R*^2^ |  | .4416  .3321 |  | <.001*** |  |  |
| *F* | 4.033 (10 and 51 DF) | | |  |  |  |
| *Residual SE* | 7.271 | | |  |  |  |
