# Supplementary material for: Happy or not? An investigative study on well-being and anhedonia in everyday life
Source: PLoS One. 2025 Sep 11;20(9):e0331769. doi: 10.1371/journal.pone.0331769 (PMC12425193; doi:10.1371/journal.pone.0331769)
Supplement: S5 Table — (DOCX) [file pone.0331769.s005.docx]

Supplementary Materials

Happy or not? An investigative study on Well-being and Anhedonia in Everyday Life

Merklein, Peterburs, Mundorf

**Table S5. Results from multiple linear regression analysis for the clinical group including psychiatric factors.** *DARS:* Dimensional Anhedonia Rating Scale; DASS: Depression Anxiety Stress Scales; MAP-SR: Motivation and Pleasure Scale - Self-Report; ISR: ICD-10-Symptom-Rating; VIF: Variance Inflation Factor.

|  | Dependent variable: anhedonia (DARS) | | | | | |
| --- | --- | --- | --- | --- | --- | --- |
|  | Unstandardized coefficients | |  |  | *Collinearity* | |
| Predictor | *b* | *SE b* | **t** | ***p*** | *Tolerance* | *VIF* |
| (Intercept) | 102.15 | 4.41 | 23.19 | <2e-16*** |  |  |
| DASS aaxiety | -1.34 | .40 | -3.32 | .001** | .341 | 2.936 |
| DASS depression | -.01 | .39 | -.03 | .974 | .162 | 6.171 |
| DASS stress | -.22 | .36 | -.62 | .539 | .350 | 2.854 |
| MAP-SR | -.70 | .18 | -3.96 | <.001*** | .446 | 2.241 |
| ISR depression | 1.87 | 2.06 | .91 | .369 | .176 | 5.670 |
| ISR anxiety | 3.20 | 1.67 | 1.92 | .061 | .339 | 2.950 |
| ISR OCD | .99 | 1.35 | .73 | .469 | .550 | 1.819 |
| ISR somatoform | 1.40 | 1.30 | 1.07 | .289 | .578 | 1.73 |
| ISR eating disorder | -.64 | 1.01 | -.64 | .527 | .705 | 1.418 |
| ISR additional scale | -.71 | 2.45 | -.29 | .774 | .335 | 2.986 |
| Psychotherapy | -.63 | 2.79 | -.23 | .822 | .592 | 1.688 |
| Psychiatric medication | 2.30 | 3.80 | .61 | .548 | .479 | 2.086 |
| Psychiatric diagnosis | .07 | 3.17 | .02 | .982 | .431 | 2.321 |
| *Multiple R*^2^  *Adjusted R*^2^ |  | .5192  .3771 |  | <.001*** |  |  |
| *F* | 3.654 (13 and 44 DF) | | |  |  |  |
| *Residual SE* | 6.354 | | |  |  |  |
